# Dextranol: An inert xeroprotectant

**DOI:** 10.1371/journal.pone.0222006

**Published:** 2019-09-06

**Authors:** Bryan J. Jones, Advitiya Mahajan, Alptekin Aksan

**Affiliations:** Biostabilization Laboratory, Department of Mechanical Engineering, University of Minnesota, Minneapolis, Minnesota, United States of America; National Cancer Institute at Frederick, UNITED STATES

## Abstract

Dextranol, a reduced dextran, prevents damage to stored dry protein samples that unmodified dextran would otherwise cause. Desiccation protectants (xeroprotectants) like the polysaccharide dextran are critical for preserving dried protein samples by forming a rigid glass that protects entrapped protein molecules. Stably dried proteins are important for maintaining critical information in clinical samples like blood serum as well as maintaining activity of biologic drug compounds. However, we found that dextran reacts with both dried serum proteins and lyophilized purified proteins during storage, producing high-molecular weight Amadori-product conjugates. These conjugates appeared in a matter of days or weeks when stored at elevated temperatures (37° or 45°C), but also appeared on a timescale of months when stored at room temperature. We synthesized a less reactive dextranol by reducing dextran’s anomeric carbon from an aldehyde to an alcohol. Serum samples dried in a dextranol-based matrix protected the serum proteins from forming high-molecular weight conjugates. The levels of four cancer-related serum biomarkers (prostate specific antigen, neuropilin-1, osteopontin, and matrix-metalloproteinase 7) decreased, as measured by immunoassay, when serum samples were stored for one to two weeks in dextran-based matrix. Switching to a dextranol-based xeroprotection matrix slightly reduced the damage to osteopontin and completely stopped any detectable damage during storage in the other three biomarkers when stored for a period of two weeks at 45°C. We also found that switching from dextran to dextranol in a lyophilization formulation eliminates this unwanted reaction, even at elevated temperatures. Dextranol offers a small and easy modification to dextran that significantly improves the molecule’s function as a xeroprotectant by eliminating the potential for damaging protein-polysaccharide conjugation.

## Introduction

Room temperature protein preservation is especially important for foodstuff, biologics, purified protein products (e.g. research, cleaning, and industrial enzymes), and clinical sample storage [1–3]. Preservation is required to avoid both chemical (e.g. oxidation) and physical forms of damage (e.g. aggregation) during storage since any damage can reduce therapeutic function, enzymatic activity, flavor, and clinical information value. Many different varieties of excipients have been employed to prevent the different forms of damage induced during processing (desiccation, freezing, etc.), and storage [4–6]. One major category of protectants used is polysaccharides; large uncharged sugar polymers, which protect proteins by a combination of mechanisms based on molecular crowding, water replacement, and glass formation [7–11].

Dextran has frequently been used as a polysaccharide xeroprotectant in dry protein formulations, mainly due to its high glass transition temperature, which facilitates room temperature storage [7, 12, 13]. Considered an inert additive [14], dextran is preferred as a protectant in pharmaceutical products [15–20]. As a result, there have been a numerous drugs on the market that contain dextran as a preservative [21, 22], including biologics [23, 24].

Dextran has one drawback, discovered by food scientists in early 1990s; storage of proteins with dextran at low-moisture conditions can facilitate formation of protein-dextran conjugates [25–28]. Conjugation of dextran with various proteins has been well documented [29–33]. Dextran is a branched D-glucose polymer by α-1,6 linkages and α-1,3 linkages at branch points with a single reducing end and multiple non-reducing ends. The conjugation is formed via a Maillard reaction between dextran’s reducing end and protein’s primary amines (N-terminus and/or lysine side chains) leading via a Schiff base to the Amadori product. While these conjugates are mostly shown to form at elevated temperatures (≥50°C) over a time period of days, there are reports of protein-sugar conjugates forming even at lower temperatures, typically over longer time scales [34–39].

Dextran-protein conjugates are larger and more soluble than the native proteins. The size of dextrans, like proteins, covers the low to high kilodalton range and thus, conjugation can easily double or triple the size of the un-modified protein. Like pegylation, dextran conjugation also increases a protein’s solubility [40] and also makes it a better emulsifier [26, 41–43]. Conjugation of carbohydrates with proteins also causes acidification (lowering of the isoelectric point) by removing positive charges from lysine residues, which Luthra and Balasubramanian [44], as well as Nacka et. al. [45], have shown to lead to protein destabilization.

Millions of clinical samples are collected each year and stored in archival biobanks for diagnostic and retrospective biomarker discovery research. The ten largest biobanks in the world collectively house approximately 35 million samples currently [46], stored in mechanical freezers or liquid nitrogen dewars. Cryogenic storage has a number of drawbacks; high cost associated with the need to maintain the cold-chain for storage and transportation of samples, and damage to samples due to freeze/thaw being the main ones [47]. These issues could be overcome by room temperature storage of the dried samples. Therefore, storage of biofluid samples (blood, urine, etc.) or extracts obtained from them (e.g. nucleic acids) in a desiccated state has emerged as an attractive alternative to cryogenic storage.

Human blood serum can be isothermally vitrified to allow room temperature storage and transportation without significant loss of biomarker information [2]. Isothermal vitrification of biofluid samples is achieved by mixing the sample usually with a sugar-based xeroprotectant cocktail, forming a glassy amorphous solid upon desiccation. The xeroprotectant cocktail we have previously developed for this purpose [2] uses a mixture of dextran, trehalose, and other low-concentration excipients. The xeroprotectant cocktail is electrospun into a microfibrous nonwoven matrix, which absorbs the biofluid sample as it dissolves in and mixes with it. We have developed this method of isothermal vitrification to ensure spatially uniform distribution of the xeroprotectant chemicals in the sample without requiring any mixing. Mixing is known to be extremely detrimental to proteins [48] while heterogeneous distribution of xeroprotectants within the sample results in inadequate stabilization [11]. Overnight desiccation of the sample-xeroprotectant mixture under vacuum then increases the glass transition temperature above 50°C enabling storage at room temperature (**S3 Fig**).

**Fig 1:**
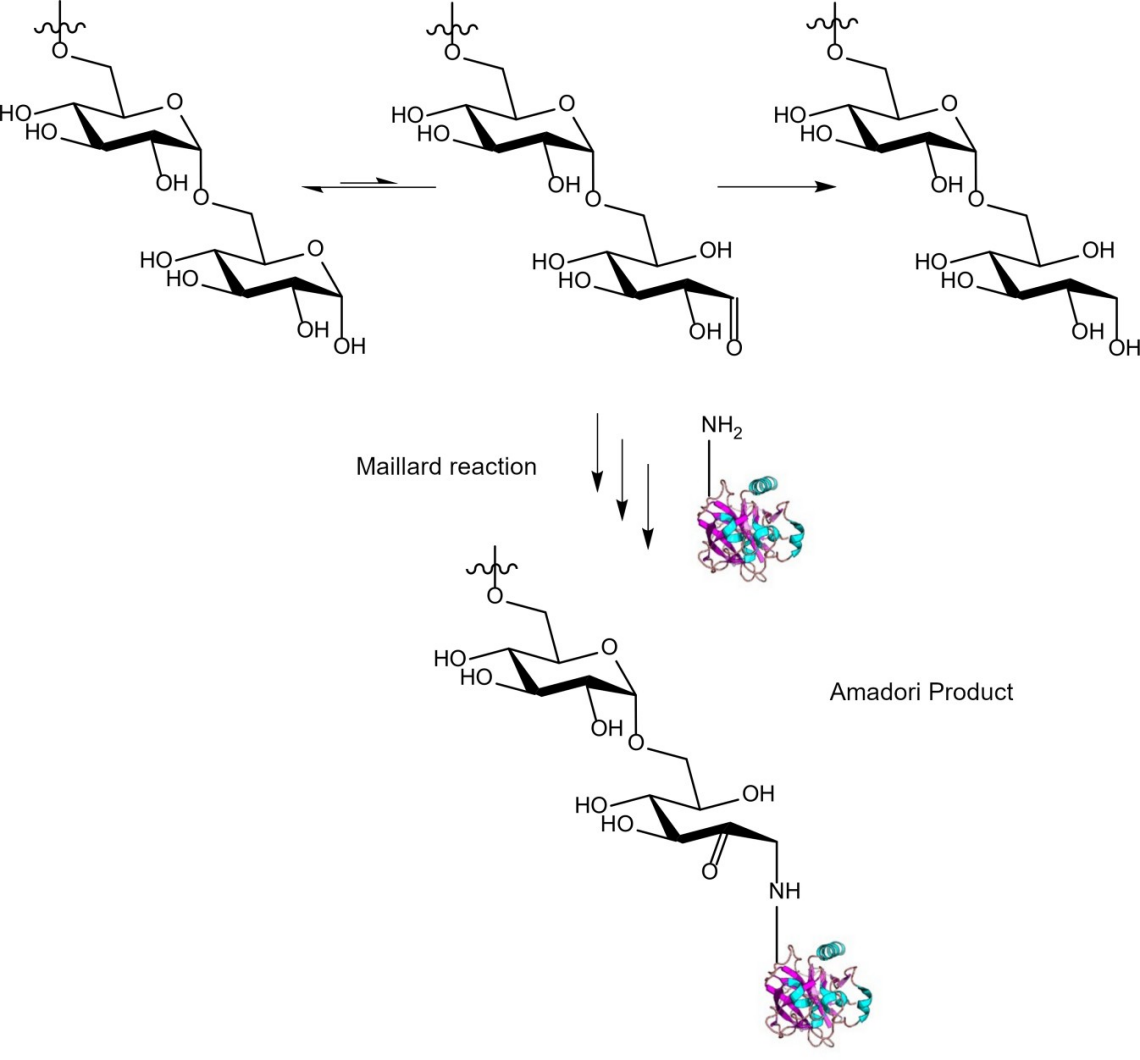
Polysaccharide modification. The cyclic and aldehyde-containing linear forms of the reducing end of a polysaccharide (e.g. dextran) are in equilibrium. The linear form is able to undergo a Maillard reaction with protein amine groups during prolonged storage or upon the addition of heat forming a glycated protein (Schiff base or Amadori Product). Reduction of the polysaccharide aldehyde to an alcohol prevents the Maillard reaction during long term storage of proteins with the polysaccharide.

Dextran plays an essential role in isothermal vitrification as it gives the dried samples the high glass transition temperature needed to maintain stability at room temperature. However, conjugation of biomarker proteins with xeroprotectants like dextran could be very detrimental to downstream analysis, especially when analytical techniques that are based on specific binding (such as ELISA) are to be utilized to detect biomarker levels or activity. In this research, we analyzed how biomarker protein conjugation with dextran during dried state storage affected detection of serum proteins post-storage. We detected the detrimental effects of dextran conjugation during prolonged storage at room temperature, 37°C, and 45°C. We found that replacing dextran in our xeroprotectant formulation with a reduced dextran (dextranol) (**Fig 1**) stabilized serum proteins more effectively, making a strong case for the use of dextranol (instead of dextran) in lyo/xero-protectant formulations to be used in biofluid sample and therapeutic protein preservation.

## Methods

### General

In the experiments conducted here, we used trehalose dihydrate (≥99% purity, Ferro-Pfanstiehl Laboratories, Waukegan, IL), dextran (35-45kDa, Sigma: D1662-500G/lot: SLBT9984), and sodium borohydride (Alfa Aesar: 88983/lot:S09D008). We purchased the other chemicals from Sigma. Human blood samples were collected from volunteers through the University of Minnesota’s (UMN) Tissue Procurement Facility (TPF) following a UMN Institutional Review Board (IRB) approved protocol (Study Number: 1011E92892). To separate serum from whole blood, we allowed whole blood to clot for at least 30 minutes and then centrifuged it for 10 min at 2000 RCF. We carefully aspirated the serum (the supernatant) at room temperature and placed it into a new centrifuge tube, taking care not to disturb the cell layer or transfer any cells. We then aliquoted the serum into microcentrifuge tubes for use in experiments.

### Dextranol synthesis

We synthesized dextranol (i.e. reduced dextran) from dextran (from *Leuconostoc mesenteroides* 35-45kDa, Sigma) using a protocol adapted from Paul et al. [49]. First, we prepared a solution of 10% w/v dextran in purified water. To this, we added 10 times molar excess of sodium borohydride. We observed mild bubbling as we stirred the solution for 20 hours, after which, we adjusted the pH down to ~5 using concentrated HCl.

To remove unreacted sodium borohydride and the byproducts, borane and NaCl, we buffer exchanged the solution into water using either dialysis (Fisherbrand 12,000–14,000 MWCO) or spin concentration tubes (Amicon concentrations 3,000 MWCO). Filtration in spin concentrators was very slow, with a flow rate of approximately 5–8 mL/hour at 4000 RCF. We repeated this process until small molecule contaminants were diluted out to less than 1%. Then, we removed water by lyophilization.

### H_1_-NMR spectroscopy

Dextran and the synthesized dextranol product were each dissolved in DMSO-d_6_ to approximate saturation. We spun tubes to remove the insoluble aggregates and added the supernatant (0.75 mL) to NMR tubes. We collected NMR spectra on Bruker 600 MHz NMR. Disappearance of anomeric proton peaks at 6.7 ppm and 6.3 ppm, corresponding to the alpha and beta stereoisomers of the anomeric center respectively, demonstrated the complete reduction to the alcohol [50].

### Production of the nonwoven xeroprotectant matrix by electrospinning

We electrospun fibers to form a dry nonvowen porous matrix from the xeroprotectant cocktail we developed. The primary components of the cocktail were dextran (or dextranol) and trehalose. Other ingredients of the cocktail included 1.5% glycerol (v/v), 1% polyethylene glycol (w/v), 0.1% Tween 20 (v/v), 0.3% gluconic acid (w/v), and 0.2% glucamine (w/v). We found that inclusion of these excipients enhanced the stability of the selected test biomarkers when desiccated [2]. In this communication, we used the same matrix formulation, except where we replaced dextran with dextranol (as described below).

To prepare the xeroprotectant cocktail, we dissolved trehalose (0.4 g/mL) and either dextran or dextranol (1 g/mL) in a solution of low concentration excipients (at concentrations mentioned above). First, we added trehalose to the solution and stirred at 200 RPM for 45 minutes to dissolve it completely. Then, we added dextran or dextranol in three stages, following each step with stirring to facilitate dissolution of the solids. We then stirred the mixture overnight (16 hours) at 200 RPM, and at 150 RPM the following day for three hours to eliminate the bubbles that formed during mixing. Finally, we allowed the solution to rest for an additional 12 hours at room temperature to ensure total dissolution. We stored the solution at 4°C when not in use.

We electrospun the xeroprotectant cocktail into microfibers across a voltage differential established in a controlled environment. We filled 1 mL syringes with the xeroprotectant cocktail and affixed a stainless steel 18-gage 0.5” long blunt-end needle. A multi-channel syringe-pump (NE-1600 multi-syringe pump; New Era Pump Systems, Farmingdale, NY) extruded the cocktail at a flowrate of 0.03 mL/min. We maintained 50% relative humidity at room temperature in an environment chamber (Electro-tech Systems, Inc., Glenside, PA). We placed the tip of the needle 15 cm away from an aluminum target and applied a voltage differential of 18 kV. These conditions were optimized to result in the most uniform electrospun fiber diameter production with optimum inter-fiber distance in the nonwoven matrix (optimized for capillary adsorption speed vs. dissolution rate) resulting in a well-controlled matrix architecture. After spinning, we desiccated the matrix in a vacuum chamber overnight to reduce the residual moisture content. We sealed and stored the electrospun xeroprotectant matrix product in a refrigerator (4°C) until needed.

### Isothermal vitrification and storage

To vitrify serum samples, we aliquoted 50 mg of electrospun matrix into round-bottom screw-top cryogenic vials. To this we added 150 μL of serum. This results in an approximate mole ratio of dextran to serum protein of 5:1 (using the average molecular weights of the dextran (40 kDa) and albumin (66 kDa) as the major protein component of serum). We dried the uncapped tubes in a vacuum chamber (at ≤ -85 kPa pressure) containing Drierite for 24 hours, reducing the water content of the dried samples to less than 20%, measured using thermogravimetric analysis, and produced a hard, glassy sample. After drying, we capped the tubes and stored them either at room temperature, or in incubators set to 37°C or 45°C for accelerated aging/high-temperature storage experiments. We also prepared biologically matched control samples of serum by freezing and storing the aliquots (without using the xeroprotectant matrix) at -20°C.

To reconstitute the vitrified samples, we added 1.5 mL PBS (Phosphate Buffered Saline) to the dried samples and incubated the tubes for 1 hour with gentle shaking, followed by gentle mixing by pipetting. This resulted in a liquid sample that was 10-fold diluted relative to the original serum. We did not reconstitute the vitrified serum samples to their original volume because this produced a very viscous and difficult to handle solution (due to the presence of the xeroprotectant sugars in the solution).

### Differential scanning calorimetry

To determine the glass transition temperature, T_g_, of vitrified samples, we conducted differential scanning calorimetry (DSC) measurements on a TA Instruments (New Castle, Delaware) DSC Q1000 V9.9 Build 303. A 2–10 mg piece of the vitrified serum in either dextran- or dextranol-based matrix was analyzed by ramping the temperature to 150°C at a rate of 10°C/minute after equilibrating at -60°C. Data were collected at 1 Hz. We analyzed the data using a custom python script that identified T_g_ as the location of the maxima of the second derivative of the heat flow data located between 20 and 80°C, and T_g,onset_ (glass transition onset) as the temperature where the heat flow deviated from its linear dependence on temperature in the glassy region by 10%. Each region was defined by a linear regression of the most linear 4°C window centered within 5°C lower (glassy region) or higher (amorphous/liquid region) than the T_g_.

### Gel electrophoresis and staining

We carried out gel electrophoresis using Invitrogen NuPAGE and NativePAGE system (ThermoFisher Scientific, Waltham, MA). For serum analysis, we loaded the equivalent of 0.4 μL of serum (i.e. 4 μL of diluted serum) per well in a 10 well gel. We prepared samples either with NativePAGE buffer for native gel electrophoresis or with LDS (lithium dodecyl sulfate) sample buffer and the reducing agent for denatured samples, and with LDS sample buffer and the reducing agent for denatured and reduced samples. We boiled both the denatured as well as the denatured and reduced samples for 10 minutes prior to loading on the gel. We ran the gels for 75 minutes across a potential difference of 150 V.

For general protein stain, we stained native gels using the NativePAGE cathode buffer per manufacturer’s instructions. We stained the denaturing gels for total protein using Imperial Protein Stain (ThermoFisher). We detected glycoproteins in gels using Pierce Glycoprotein Staining kit (Pierce #24562, lot#SK258276) per manufacturer’s instructions.

### ELISA analysis

We performed enzyme-linked immunosorbent assays (ELISAs) for osteopontin, MMP-7, neuropilin-1, or prostate specific antigen (PSA), following manufacturer’s instructions. We used the following kits: Human Osteopontin ELISA Kit (Abcam, Cambridge, UK, #ab192143), Human Neuropiln-1 ELISA Kit (Abcam #ab227901), Human Total Prostate-Specific Antigen ELISA Kit (Abcam #ab188388), and Quantikine ELISA Human Total MMP-7 (R&D Systems Minneapolis, MN, #DMP700). Plates were read in a Tecan Infinite 200 Pro M Nano plate reader at 450nm.

### TCA precipitation

We carried out TCA precipitation by mixing equal parts of resuspended vitrified serum (or the frozen serum control, equivalently diluted after thawing) and 20% TCA (trichloroethanoic acid). After mixing 100 μL of each part, we incubated on ice for 15 minutes. Subsequently, we centrifuged 10 minutes at 10,000 RCF, photographed the tubes, and decanted the supernatant. The pellets were suspended in 400 μL saturated guanidine hydrochloride for protein quantification.

### Protein quantification

Amount of protein in the sample was quantified using BCA Protein assay (ThermoFisher). The standard curve was constructed using bovine serum albumin (BSA). Samples used in TCA precipitation were analyzed both in the “soluble” fraction and the “precipitate”. Assay was quantified using a Tecan Infinite 200 Pro M Nano plate reader at 562 nm.

### Lyophilization

As an analog to biological drug formulations IgG was lyophilized in either dextran or dextranol. We prepared a solution of IgG (2 mg/mL, Human IgG Isotope Control from Invitrogen, product #02–7102, supplied at 5 mg/mL in PBS) and dextran or dextranol (20 mg/mL). Solutions were aliquoted (200 μL) into glass vials, frozen at -80°C and then dried under vacuum (~0.05mBar) for 24 hours. Vials were then capped and stored at either room temperature or 45°C.

### Size exclusion chromatography

Size exclusion chromatography was carried out to quantify large-molecular-weight species in stored samples. We performed size exclusion chromatography on a HiLoad 16/600 Superdex 200 pg column on an ÄKTAexplorer instrument (GE Life Sciences). The mobile phase was TrisHCl (20mM) with sodium chloride (500mM) at pH 7.4. Flow rate was set to 1 mL/min and elution was detected by absorbance at 280 nm. Lyophilized samples were reconstituted in 200 μL of the mobile phase buffer. After allowing 20 minutes to dissolve, 150 μL of the sample was diluted with 600 μL mobile phase. This was centrifuged at ≥10k RCF for 10 minutes to remove any insoluble protein or debris. Then 700 μL of the solution was loaded onto the column after equilibrating for at least one column volume (120 mL). The void volume of the column was 40–45 mL. Native IgG peak was detected between 58 and 72 mL, while a high-molecular-weight peak/shoulder was detected between 50 and 58 mL. Data were integrated and graphed using Microsoft Excel and a custom Python script. Percentage of high-molecular-weight species in the solution was calculated as the integrated area of the 50–58 mL peak divided by the integrated areas of both the native and high-molecular-weight peaks.

## Results

**Fig 2:**
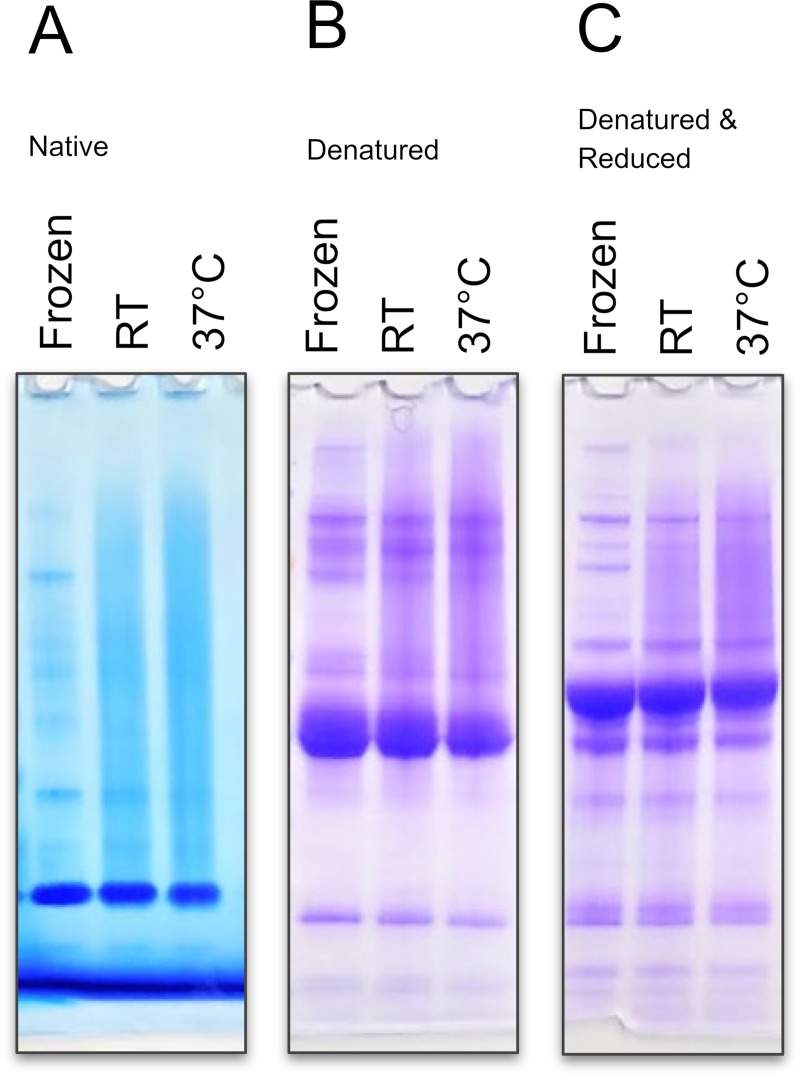
High molecular weight smearing appears in vitrified samples. Human serum after 16 weeks of storage after isothermal vitrification using dextran-based xeroprotectant matrix show high molecular weight smearing. Vitrified samples were either stored at room temperature (RT) or 37°C and reconstituted in PBS. Frozen samples were kept at -20°C. Gel electrophoresis was carried out using (Panel **A**) non-denaturing conditions, (Panel **B**) denaturing conditions, or (Panel **C**) denatured & reducing conditions. High molecular weight smears were present in all vitrified samples stored for 16 weeks and were more pronounced in samples stored at the higher temperature. Smearing did not disappear upon denaturation or reduction.

When the dextran-based xeroprotectant matrix was used to preserve blood serum by isothermal vitrification, after 16 weeks of storage high-molecular weight smearing appeared in electrophoresis gels. While freshly vitrified (and immediately reconstituted) serum appeared identical to fresh and frozen serum (-20°C) on gel electrophoresis, smearing in the vitrified samples was very prominent (**Fig 2**), especially when the samples were stored at elevated temperatures. Individual protein bands were less detectable due to decreased intensity and smearing. While smearing was worse in native protein gels (Panel A in **Fig 2A**), denatured (Panel B in **Fig 2**), and denatured & reduced (Panel C **Fig 2**) samples also showed smearing. This indicates that the observed smearing is not solely due to non-covalent or disulfide cross-linked aggregated proteins. Non-covalent aggregates would be broken apart by the boiling in LDS (lithium dodecyl sulfate) sample buffer and thus would not be present in the denatured gels. Similarly, disulfide cross-linked aggregates would be broken by the reducing agent DTT (dithiothreitol) present in the denatured and reduced gel; yet the smearing remained. This indicated that the observed smearing is due to a significant, non-disulfide-bonded, covalent modification of proteins (potentially by dextran in the matrix).

**Fig 3:**
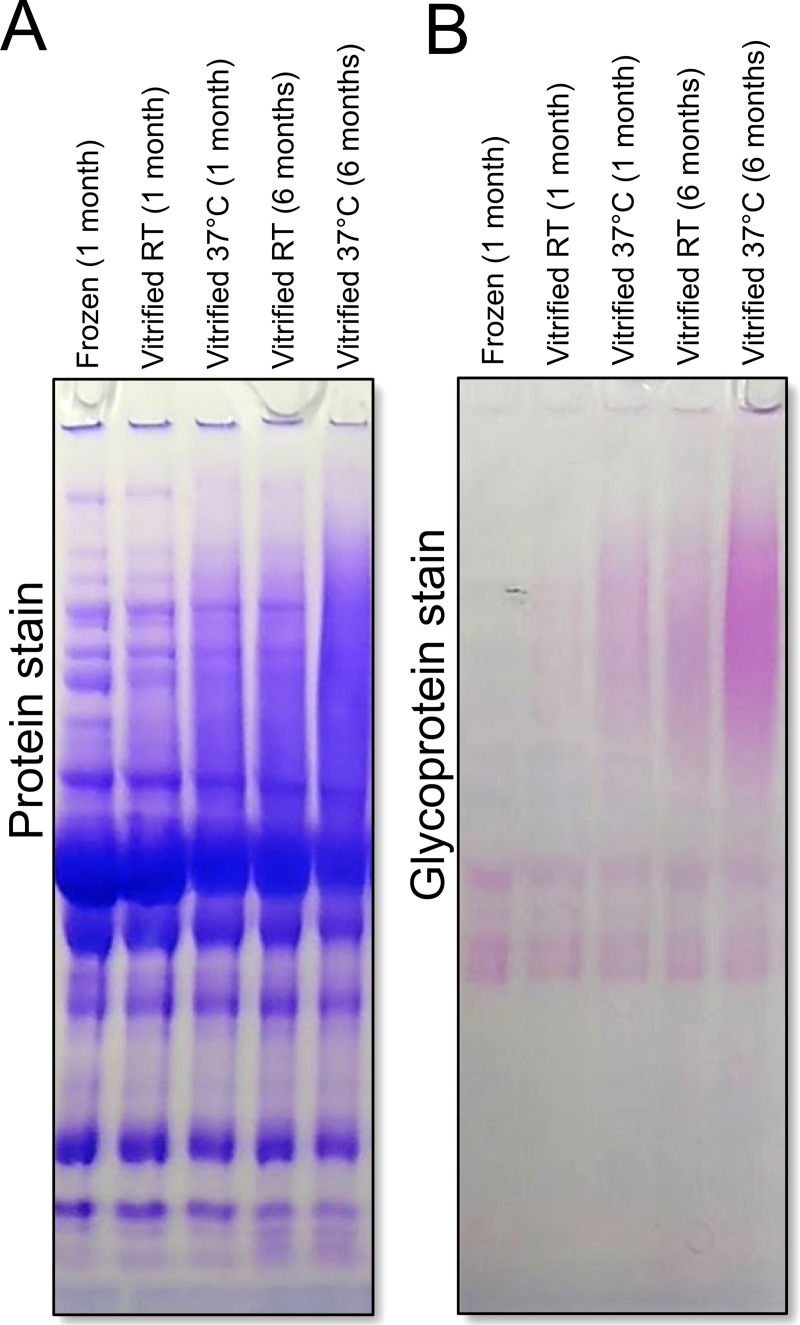
High molecular weight smearing worsens over time and at elevated temperature storage, and stains positive for glycosylation. Serum samples were vitrified in dextran-based xeroprotectant matrix and stored for 1 or 6 months at either room temperature (RT) or 37°C. Samples were run on SDS-PAGE under denaturing/reducing conditions and stained (Panel **A**) for total protein or (Panel **B**) for glycoprotein. Smearing worsened with higher temperature storage and with increased storage time. Glycoprotein stain indicates high-molecular weight smears are glycosylated; suggesting covalent attachment of dextran to proteins.

High molecular weight smearing and glycosylation increased both with storage time and storage temperature and were accompanied by increased solubility. Smearing was faint after only 1 month of storage at room temperature, but increased when either storage temperature was increased to 37°C or storage time was extended to six months (Panel A in **Fig 3**). These effects were additive as after six months of storage at elevated temperature, the smearing and decrease in contrast in individual protein band was significantly worse than storage either for six months at room temperature or for one month at elevated temperature. Concurrent with the increase in smearing was the increased glycoprotein staining in the high molecular weight region (Panel B in **Fig 3**). This indicates that high-molecular weight bands in the smear have been glycosylated. We also noticed that the vitrified samples that had become more soluble and would not precipitate efficiently when a standard TCA precipitation protocol was used (**S1 Fig, S1 Table**).

**Fig 4:**
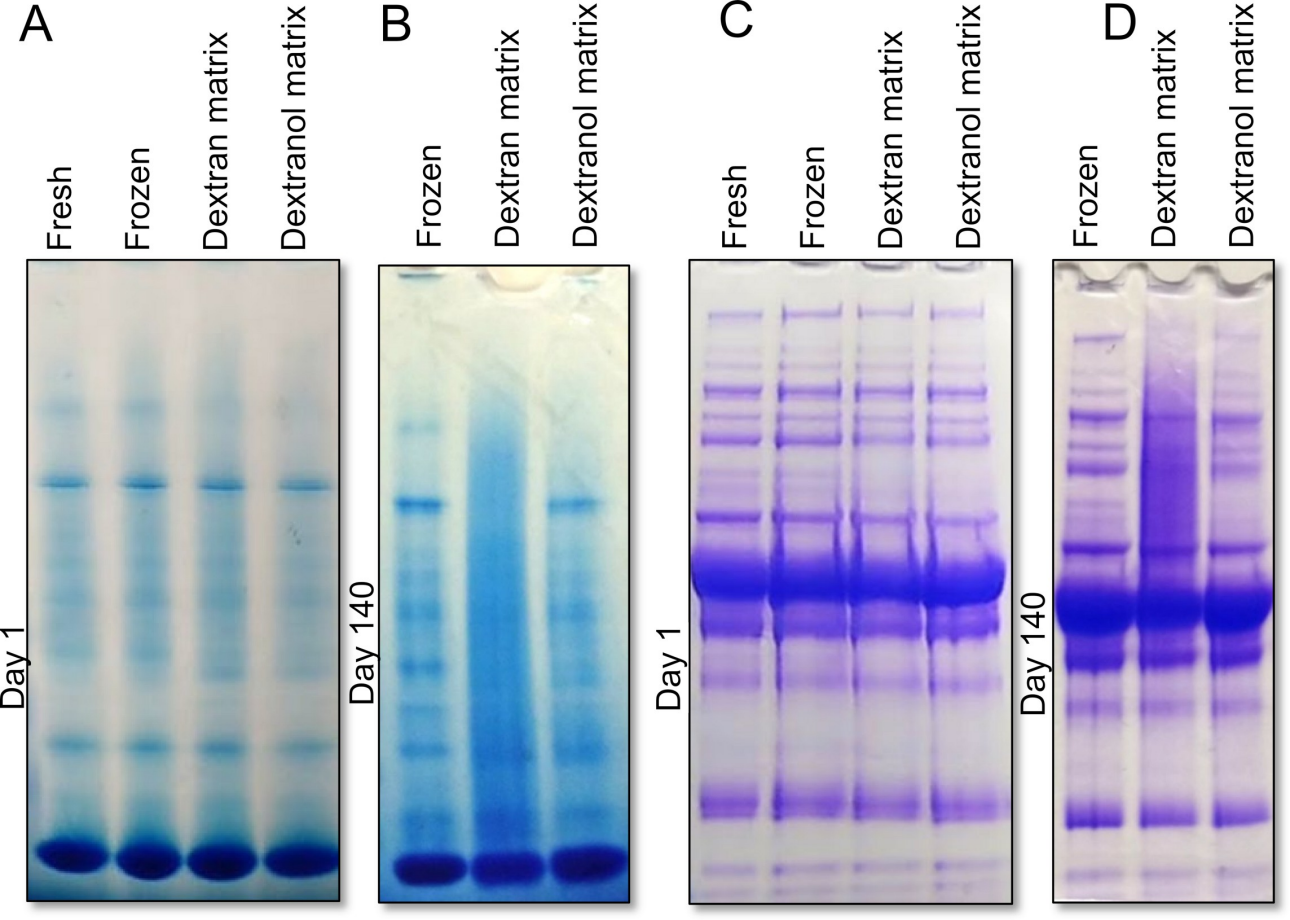
Dextranol-based xeroprotectant matrix preserves proteins and prevents smearing. Serum samples were either fresh, frozen, or vitrified in either a dextran-based or dextranol-based xeroprotectant matrix. Serum was analyzed (Panel **A** and **C**) immediately after vitrification (Day 1) or (Panel **B** and **D**) after storage for 140 days at 37°C. Vitrified samples were reconstituted in PBS. Gel electrophoresis was carried out under (Panel **A** and **B**) native conditions, and (Panel **C** and **D**) denaturing/reducing conditions. After two weeks, smearing was visible in samples preserved in dextran-based matrix, but not in dextranol-based matrix. Gel images obtained on intermediate time points can be found in **S4 Fig**.

Non-reducing dextran, i.e. dextranol, based xeroprotectant matrix, on the other hand, was able to better preserve serum proteins during extended storage. We reduced the reducing end of dextran from an aldehyde to a more inert alcohol and verified the complete reaction by observing loss of anomeric proton peaks in H_1_-NMR spectra (**S2 Fig**). Whereas vitrifying serum using a dextran-based xeroprotectant matrix resulted in significant high-molecular weight smearing, serum vitrified in dextranol-based xeroprotectant matrix did not have any smearing (**Fig 4 and S4 Fig**). Samples that were frozen, vitrified in dextran-based matrix or dextranol-based matrix (after 1 day of storage) were all virtually indistinguishable from fresh, never frozen serum when analyzed by gel electrophoresis under either native (Panel A in **Fig 4**) or denaturing conditions (Panel C in **Fig 4**). However, after 140 days of storage at 37°C, samples vitrified in dextran-based matrix showed significant smearing, while dextranol-based matrix samples were almost indistinguishable from frozen or fresh serum.

Storage at higher temperatures allowed us to simulate long-term storage at room temperature. Like samples vitrified in dextran, samples vitrified in dextranol also form a glassy state with a glass-transition above 50°C (**S3 Fig**). The glass-transition temperature of the vitrified serum in either dextran or dextranol-based matrix was 50–55°C (**S3 Fig**). Therefore, in all storage experiments, the storage temperatures were kept below 50°C. While decay reactions do not uniformly scale with temperature, we expect a doubling in degradation rates every 5–10°C increase in temperature [51]. The activation energy of Maillard reactions between reducing sugars and proteins have been reported over a wide range, but are typically found to be about 100 kJ/mol [52]. Using this value in the Arrhenius equation, we estimated a doubling effect every 5°C. Thus 37°C storage corresponded to approximately six times faster aging of the samples (as compared to room temperature storage) while 45°C storage corresponded to approximately seventeen times faster aging.

**Fig 5:**
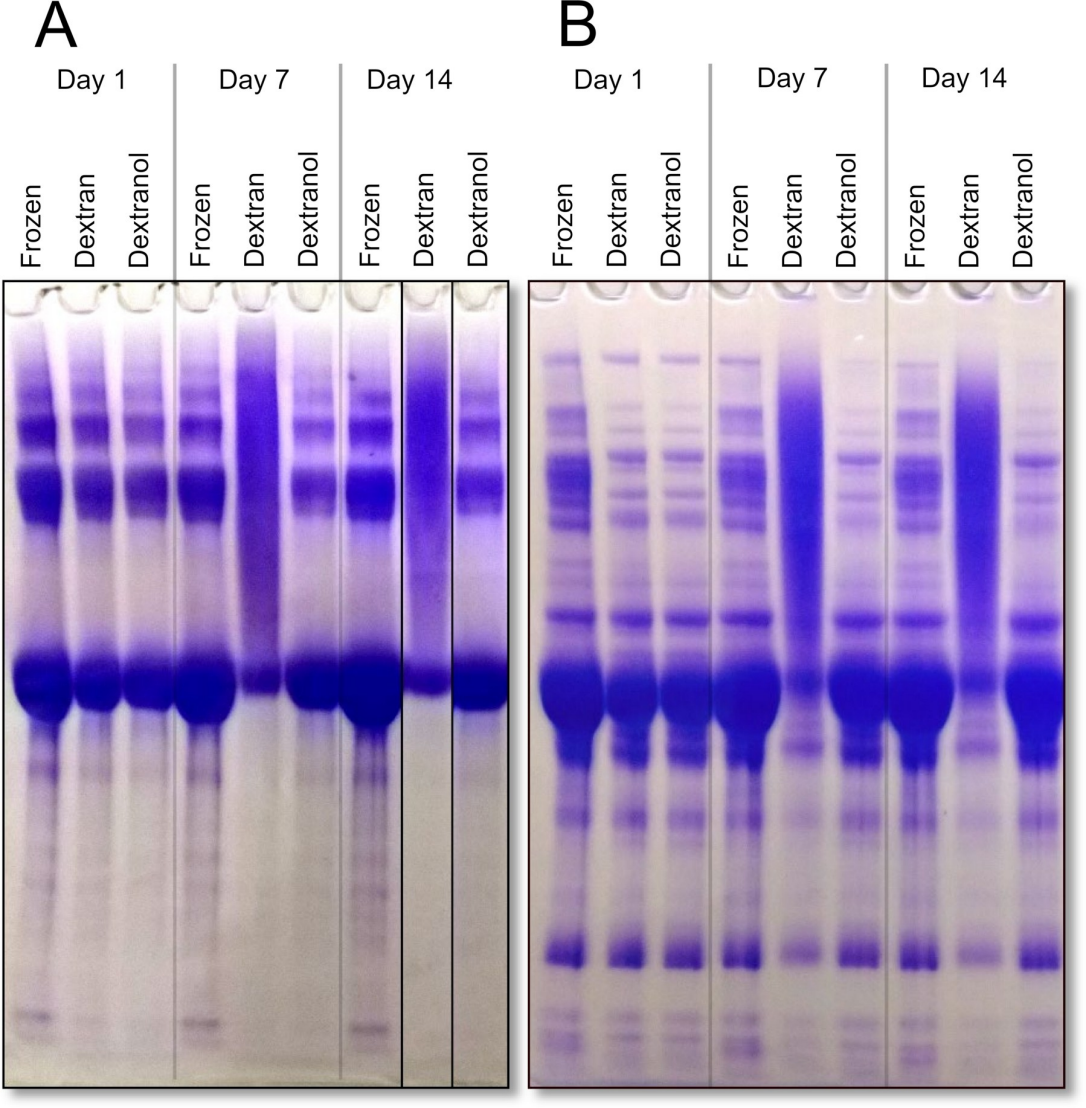
Dextranol-based xeroprotectant matrix protects human serum proteins and BSA when stored at 45°C. Coomassie stained gel showing results of high temperature storage of (Panel **A**) BSA (Bovine serum albumin) and (Panel **B**) human serum. Immediately after vitrification, samples preserved in dextran and dextranol-based xeroprotectant matrices looked similar to frozen sample (left). After 7 days (mid), and 14 days (right) BSA stored in dextran-based matrix was mostly in a high-molecular weight smear. BSA stored in dextranol-based matrix still resembled the frozen sample. Note that it is common to see higher molecular bands in SDS-PAGE of BSA due to irreversible multimer formation [53, 54].

Dextranol also protected isothermally vitrified serum or an isolated protein sample when stored at high temperatures (45°C). To examine the storage stability of a purified protein product we selected BSA as a model protein. BSA vitrified in dextran-based matrix immediately after desiccation looked identical to a frozen control, but after storage for 1–2 weeks at 45°C, the main BSA monomer band was highly diminished, and replaced by a high-molecular weight smear (Panel A in **Fig 5**). In the dextranol-based matrix, this kind of damage was absent; the sample stored at 45°C for two weeks was indistinguishable from the freshly vitrified sample and was very similar to the frozen sample.

In addition to stabilizing BSA, the dextranol-based matrix also protected human serum proteins when isothermally vitrified and stored at higher temperature. Most protein bands became largely diminished when the sample was stored for one or two weeks in a dextran-based matrix (Panel B in **Fig 5**), and were replaced by a high-molecular weight smear, but storage in dextranol-based matrix protected the proteins. The serum proteins look indistinguishable from freshly vitrified serum after 2 weeks at 45°C, and looked very similar to frozen serum.

In addition to providing overall protection to high abundance serum proteins, and albumin specifically, we also found that dextranol-based xeroprotectant matrix provided much better protection than the dextran-based matrix for four individual proteinaceous biomarkers in the high-temperature stored serum samples. We selected 4 cancer biomarkers to be tested for stability. All four biomarkers that were examined (prostate specific antigen, neuropilin-1, osteopontin, and matrix-metalloproteinase 7) showed losses (as measured by ELISA) after one or two weeks of storage at 45°C (**Table 1** and **S5 Fig**). In dextranol-based matrix, PSA (Panel A in **S5 Fig**) and neuropilin (Panel B in **S5 Fig**) levels on day 1 were slightly reduced, potentially due to the desiccation process (by 7 and 8%, respectively), but were stable during seven or fourteen days of storage. MMP-7 levels (Panel D in **S5 Fig**) in dextranol-based matrices remained slightly above the frozen control throughout this experiment. However, osteopontin levels in the dextranol-based matrices fell in a similar pattern to that seen in dextran-based fibers, although not quite to the same extent.

**Table 1:** Biomarker preservation after 14 days storage of vitrified samples at 45°C^a^.

Fibers	Osteopontin	Neuropolin	MMP-7	PSA
Dextran^b^	61% ± 1%	39% ± 1%	82% ± 4%	69% ± 4%
Dextranol^c^	77% ± 1%	87% ± 3%	107% ± 3%	94% ± 3%

^a^ Amount of indicated protein as a percentage of frozen sample without xeroprotectants, ± the standard deviation of three technical replicates.

^b^ Serum vitrified in dextran-based matrix.

^c^ Serum vitrified in dextranol-based matrix.

Lyophilization is the method of choice for stabilization of pharmaceutical proteins. In lyophilization, the samples are first frozen and then desiccated by applying vacuum, as opposed to isothermal vitrification, where samples are desiccated at room temperature without freezing. Antibodies like IgG are one of the most common biologic drugs. To test whether formation of dextran-protein conjugates could also occur in biologic drugs, we lyophilized IgG with either dextran or dextranol and examined the formation of high-molecular-weight bands after storage. After one month of storage, IgG remained almost exclusively in its native form (**Table 2**, **S6 Fig** green shaded area) when frozen or lyophilized with dextranol, but high-molecular-weight species (**Table 2**, **S6 Fig** sand colored shaded area) increased when lyophilized with dextran, especially for samples stored at high temperature. IgG had less than 10% high-molecular-weight species when lyophilized with dextranol or stored frozen (**Table 2**), but when lyophilized with dextran, after one month at room temperature, fraction of high-molecular-weight species increased to 14%. In high temperature (45°C) stored samples, the fraction of high-molecular-weight species increased to 39%.

**Table 2:** High-molecular-weight adduct formation in lyophilized IgG.

Storage Condition^a^		High-molecular-weight species^b^
Temperature	Xeroprotectant	
-20°C	None	9%
45°C	Dextran	39%
	Dextranol	9%
Room Temperature	Dextran	14%
	Dextranol	8%

^**a**^ Samples stored for either 30 days (45°C samples) or 33 days (room temperature and -20°C samples). Before lyophilization, samples contained 2 mg/mL IgG with 20 mg/mL of the indicated xeroprotectant.

^**b**^ high-molecular-weight adducts were quantified by comparing the area of the high-molecular-weight species to the total area of both high-molecular-weight and native IgG peaks by size exclusion chromatography.

Additionally, MALDI-TOF mass spectrometry was conducted on lyophilized protein-dextran/dextranol formulation (**S7 Fig**). We used myoglobin (M_w_ = 19.3kDa) in these experiments to ensure that high molecular weight adducts could still be detectable. The samples were stored either in -20°C or at 45°C for three weeks. A clear spectrum could be obtained from the fresh protein in solution (positive control) (Panel A in **S7 Fig)**. A peak can be seen at approximately 19.3kDa, which is the theoretical molecular weight of recombinant human myoglobin. Additionally, peaks can be observed at 38.6kDa, and 57.9kDa, possibly corresponding to the dimer, and trimer forms of the protein, respectively. Spectra from thawed protein-dextran, and protein-dextranol solutions can be seen in Panel B and C in **S7 Fig** respectively. In both spectra, the protein peak can be seen around 19.5kDa. The dimer and the trimer peaks of the protein are largely reduced. Yet, in both spectra, a peak can be observed around 40kDa, at the average molecular weight of dextran and dextranol we used in this study. While this peak overlaps with the position of the possible dimer peak observed before, it is broader and of higher molecular weight. The spectra for lyophilized samples stored at 45ºC can be seen in Panel D and E **S7 Fig**. In the lyophilized protein-dextran sample (Panel D in **S7 Fig**), neither protein nor dextran peaks can be detected. High-molecular weight moieties like dextran-protein adducts as well as aggregation of protein can cause difficulty in ionizing proteins (due to their increased size) and therefore, cannot be detected through MALDI-TOF MS. While MS was not able to directly detect high-molecular weight species that were seen by SDS-PAGE and SEC, it does support a mode of damage due to dextran’s reducing aldehyde. In the protein samples lyophilized with dextranol (Panel E in **S7 Fig**) protein and dextranol peaks can be detected after reconstitution, thus suggesting that dextranol does not form the high-molecular weight adducts or aggregates as easily as dextran.

## Discussion

Our results show that dextran is not a good xeroprotectant for proteinaceous biomarkers, as it leads to the formation of dextran-protein conjugates in the dried state. Conjugation is likely due to a Maillard reaction forming an Amadori product between the primary amines of a protein (lysine residues and the N termini) and the reducing aldehydes of the dextran chain. In our isothermal vitrification formulation molar concentrations of the reactants are within an order of magnitude, with a molar excess of dextran molecules to protein molecules, but a molar excess of protein amine groups to dextran reducing groups. While our experiments show this reaction happens to a large extent in a matter of days during storage at higher temperatures (37°C or 45°C), even during room temperature storage, the product is readily apparent after four months. The addition of dextran to proteins was determined to affect their physical properties like solubility as well as their antigenicity (antibody reactivity) as seen by decreased reactivity with ELISA antibodies. It is well known that conjugating polymers to proteins like polyethylene glycol or polysaccharides can cause large changes in antigenicity [55, 56]. Decreased antigenicity results in less accurate immunoassays, which is a problem for detecting biomarkers in stored biospecimens. We found that dextran-protein adducts form both in isothermally vitrified complex samples (such as the blood serum) as well as samples of pharmaceutical relevance such as lyophilized IgG. This damage is concerning since dextran is currently used as a xeroprotectant in a number of lyophilized pharmaceutical formulations [21–24], including formulations for vaccines [24] where decreased antibody reactivity would cause reduced efficacy.

Conjugation potential of dextran was well known to food scientists however, this knowledge did not translate into the pharmaceutics field. Many studies, despite observing protein damage in the presence of dextran, did not identify the mechanism of the damage as conjugation. Lyophilization research that focused on therapeutic proteins established that formulations containing dextrans cause increase in protein size [57–63], increases solubility [64], and altered acidity [65], but this was interpreted as aggregation or damage, and not attributed to dextran-protein conjugation. Studies using size exclusion chromatography observe increased protein size after storage with dextran and speculate that this is caused by “soluble aggregation” [57–63]. However, size exclusion chromatography cannot distinguish dextran conjugated proteins from dimeric or oligomeric protein “aggregates”. We replicated the appearance of high-molecular-weight species in size exclusion chromatography of proteins lyophilized in dextran (**S6 Fig** and **Table 2**). We found that replacing dextran with the inert dextranol prevented the formation of these high-molecular-weight species, demonstrating that the “soluble aggregates” reported were likely primarily dextran conjugates (potentially, in addition to protein self-aggregation). Additionally, mass spectrometry was employed to study the formation of these dextran conjugates. While protein samples were detected in the protein lyophilized with dextranol, no protein could be detected in the lyophilized protein-dextran formulation. This could be due to the formation of the high-molecular weight species, which have difficulty ionizing due to their large size [66–68]. Similarly, studies that used dextran as a xeroprotectant that reported increased solubility [64] and acidity [65] of the preserved proteins could also have observed the effects of dextran conjugation. It is known that the degree of dextran modification varies between proteins [25, 26]. This is likely, in part, due to differences in the number and position of surface lysines.

Specifically, Yoshioka et al. characterize dextran mediated damage of lyopholized beta gamma globulin as denaturation and/or aggregation, yet their results are more consistent with dextran-protein conjugation than simple denaturation or aggregation [57]. They conducted experiments with different sizes of dextrans (10kDa-510kDa) and reported that at constant weight fraction in solution, smaller size dextrans (i.e. higher numbers of reactive aldehyde ends per gram of dextran in the solution) caused more damage. They proposed that “the effect of the molecular weight of dextran on the protein stability … could be explained in terms of the parameters obtained by ^1^H-NMR such as T_mc_ [molecular mobility changing temperature]”. A simpler explanation is that over 50-fold variation in molarity of the reactive aldehyde groups, between the smallest and the largest dextrans, is the main cause. Yoshioka et al. also observed that the increases in sizes of the damaged protein as measured by size-exclusion chromatography correlated with the molecular weight of the dextran; a result that would not be expected by just denaturation and aggregation, but would be expected if the “damaged proteins” they observed were in fact protein-dextran conjugates.

Qi and Heller, also found evidence for dextran modification of proteins without identifying it as such [15]. They report that in liquid state storage, dextran damaged therapeutic peptide insulinotropin. This damage was different from that observed in the absence of dextran and could be prevented by adding certain excipients into the solution (sodium metabisulfate or amino acids) that could react with dextran’s aldehyde group.

Pikal et al. also found evidence for dextran-protein conjugation, which they characterized as soluble “aggregates” [59, 60]. When they stored human growth-hormone freeze-dried in dextran, it became larger as measured by size-exclusion chromatography. The increase in the size of the protein in the presence of dextran was greater than when it was lyophilized in its absence or with excipients like glycine, mannitol, hydroxyethyl starch, or trehalose. Interestingly, Pikal et al. suggest that the damage observed when the protein was stored with lactose was due to protein-sugar conjugates formed via a Maillard reaction, yet they did not propose the same mechanism for dextran-mediated damage.

Other researchers in pharmaceutical field have looked at intentionally covalently attaching dextran to drug molecules [69]. This has been shown to have numerous effects on a drug’s function. Some of the effects are desired; such as improved on-target efficacy [70], reduced toxicity [71], and increased stability [72]. However others found detrimental effects from conjugating drugs with dextran, including decreased activity [72] and in one case, a dextran drug conjugate (dextran-doxorubicin) failed in a phase one clinical trial due to higher hepatotoxicity than unmodified doxorubicin [73].

We found that a small chemical modification, converting dextran to dextranol, eliminates this unwanted reaction and better stabilizes human serum proteins during storage. The reduction of dextran’s terminal aldehyde to an alcohol, forming dextranol, ensures inertness. This relatively small chemical modification on a large (~40 kDa) dextran monomer produced a new xeroprotectant that has all the desirable physical properties of dextran without the potential for the detrimental Maillard reaction. Dextranol has been synthesized before to enhance iron crystal growth and the properties of iron colloids [49, 74]. However, this communication is the first report on the use of dextranol as an effective xeroprotectant.

Unlike dextran, dextranol-based xeroprotectant matrix we developed was able to protect serum proteins during extended storage with little damage. After 140 days at 37°C or 14 days at 45°C serum proteins stored in dextranol-based matrix showed little sign of degradation by gel electrophoresis, while proteins in dextran-based matrix largely reacted with dextran, visible as high-molecular weight smearing on gels. ELISA analysis of four selected biomarkers showed that detected levels of three of the four biomarkers did not decrease over storage at 45°C when stabilized using the dextranol-based matrix, while levels of all four fell significantly when stored using the dextran-based matrix. Even osteopontin, the one biomarker that did degrade during storage in the dextranol-based matrix, did not lose activity as much as it did in the dextran-based matrix. It is yet unknown what the persisting mechanism of damage was in this case. Osteopontin is frequently cleaved into smaller form, often by thrombin [75]. If this were happening in the vitrified sample it may show up as loss of osteopontin since the ELISA antibodies were developed against full-length osteopontin [76]. While it is known that inherently disordered proteins like osteopontin [77] are more prone to degradation intracellularly [78], it is not known if that is also the case extracellularly.

While intentional conjugation of proteins with dextran can have useful applications by improving protein solubility and heat stability [30] or as improved food emulsifiers [25], conjugation will inevitably change the physical and chemical properties of the proteins, which can cause loss of function and loss of detection in clinical assays. Other carbohydrates; including lactose, maltodextrin, glucose, and galactomannan; are also known to similarly react with proteins over extended storage times [34–36, 39, 79, 80]. Thus, the use of dextran, or other reducing carbohydrates, as xeroprotectant agents, especially for prolonged times or at higher temperatures should be avoided. The modified sugar polymer dextranol is an attractive xeroprotectant since it provides the glass-forming protection of dextran while avoiding the damaging Maillard reaction.

## Supporting information

S1 Table Vitrified serum stored 35 days in dextran-based matrix is not effectively precipitated by TCA. ^a^ Whole protein concentration after resuspension of vitrified samples and thawing of frozen samples. ^b^ Protein remaining following resuspension after TCA precipitation. ^c^ Undiluted serum protein concentration reported. ^d^ Serum frozen and stored at -20°C. ^e^ Serum vitrified in dextran-based matrix and stored at room temperature. ^f^ Serum vitrified in dextran-based matrix and stored at 37°C.
(DOCX)

S1 Fig TCA precipitation of serum proteins stored 35 days in dextran-based matrix. Diminished pellet size shows increased solubility somewhat in the sample stored at room temperature (middle tube) and largely in the sample stored at 37°C (right tube) compared to frozen sample without matrix (left tube).
(DOCX)

S2 Fig H_1_-NMR showing complete reaction and disappearance of protons attached to the anomeric carbon. (**A**) Proton signals at (6.74 and 6.30 ppm) indicate dextran aldehydes, while (**B**) loss of these signals shows that aldehydes were completely reduced to alcohols in dextranol.
(DOCX)

S3 Fig T_g_ and T_gon_ of serum preserved in either dextran and dextranol. DSC traces of serum samples preserved in either dextran (**A**) or dextranol (**B**) based matrix after 30 days of storage at room temperature. Solid blue line is DSC data, dashed green line is linear glass-transition fit, dashed red line is the linear fit of liquid region (fit to grey-shaded region), dashed cyan line is the liner fit of the glassy region (fit to green-shaded region). The vertical tan line marks the glass transition temperature (T_g_) at 54.7°C (dextran, **A**) and 53.9°C (dextranol, **B**). The vertical gold line marks the glass transition onset temperature (T_gon_) at 53.2°C (dextran, **A**) and 53.1°C (dextranol, **B**).
(DOCX)

S4 Fig Dextranol preserves proteins and prevents smearing compared with dextran. Serum samples were either fresh, frozen, or vitrified in either a dextran-based or dextranol-based xeroprotectant matrix. Serum was analyzed after 1, 7, 14, 28, 60, and 140 days at 37°C (data from days 1 and 140 in **Fig 4**). Vitrified samples were reconstituted in PBS. Gel electrophoresis was carried out under both native conditions (**A**) and denaturing/reducing (**B**) conditions. A duplicate denatured/reduced gel from the sixty-day old sample was also stained for glycoproteins (**C**). After two weeks, smearing is visible in samples preserved in dextran, but not in samples preserved in dextranol.
(DOCX)

S5 Fig Serum biomarker levels are better retained after storage at 45°C when vitrified in dextranol than dextran. ELISA analysis of biomarker stability in vitrified human serum stored at high temperature (45°C), preserved in either dextran-based (orange) or dextranol-based matrix (green). Four biomarkers examined were (**A**) PSA (prostate specific antigen), (**B**) neuropilin-1, (**C**) osteopontin, and (**D**) MMP-7 (matrix-metalloproteinase 7). Serum samples were analyzed immediately after desiccation (day 1), and one week and two weeks after desiccation and storage. Values are normalized to biomarker content in frozen control samples. Error bars are standard deviation of three replicates.
(DOCX)

S6 Fig High-molecular-weight adducts of lyophilized IgG by size-exclusion chromatography at multiple storage time points. Comparison of lyophilized and frozen samples after indicated storage duration and temperature. Lyophilized samples were preserved with either dextran or dextranol while the frozen sample contained neither xeroprotectants. After reconstitution or thawing, native IgG (green colored area) and soluble large-molecular-weight species (sand colored area) were distinguishable size-exclusion chromatography.
(DOCX)

S7 Fig Mass Spectrometry on Lyophilized Myoglobin with Dextran/Dextranol. MALDI-TOF MS was performed on myoglobin lyophilized with dextran or dextranol (in PBS solution). A clear spectrum for fresh protein in solution (positive control) can be seen in **A**. Spectra for frozen dextran and dextranol can be seen in **B,** and **C,** respectively. The spectra for lyophilized samples stored at 45ºC can be seen in **D,** and **E,** respectively.
Mass Spectrometry Method: Recombinant human myoglobin (Novus Biologicals) was mixed with dextran/dextranol in PBS solution at final concentration of 6μM and 500μM respectively. 200 μL aliquots were lyophilized and either stored frozen at -20°C or stored at 45°C for three weeks. The frozen and vitrified samples were reconstituted in 100μL DI water post storage. A fresh control samples containing myoglobin in PBS was also prepared. The Center of Mass Spectrometry and Proteomics at the University of Minnesota performed matrix assisted laser desorption/ionization-time of flight (MALDI-TOF) mass spectrometry (MS) on these samples. The samples were prepared using C4 ZipTip protocol prior to loading them on Bruker’s Autoflex speed MALDI-TOF System. The ionization matrix was Sinapinic acid (SA) for the control sample and super-dihydroxybenzonic acid(sDHB) matrix for dextran/dextranol containing samples, where it was found to provide better signal than SA. The data was analyzed using mMass software.
(DOCX)
