# Supplementary material for: Dextranol: An inert xeroprotectant
Source: PLoS One. 2019 Sep 6;14(9):e0222006. doi: 10.1371/journal.pone.0222006 (PMC6730909; doi:10.1371/journal.pone.0222006)
Supplement: S1 Table — a Whole protein concentration after resuspension of vitrified samples and thawing of frozen samples. b Protein remaining following resuspension after TCA precipitation. c Undiluted serum protein concentration reported. d Serum frozen and stored at -20°C. e Serum vitrified in dextran-based matrix and stored at room temperature. f Serum vitrified in dextran-based matrix and stored at 37°C. (DOCX) [file pone.0222006.s001.docx]

**S1 Table. Vitrified serum stored 35 days in dextran-based matrix is not effectively precipitated by TCA.**

|  | Whole^a^ | Precipitated^b^ |
| --- | --- | --- |
|  | mg/mL^c^ | mg/mL^c^ |
| Frozen^d^ | 59 | 48 |
| Vitrified (RT)^e^ | 60 | 46 |
| Vitrified (37º C)^f^ | 58 | 12 |

^a^ Whole protein concentration after resuspension of vitrified samples and thawing of frozen samples. ^b^ Protein remaining following resuspension after TCA precipitation. ^c^ Undiluted serum protein concentration reported. ^d^ Serum frozen and stored at -20°C. ^e^ Serum vitrified in dextran-based matrix and stored at room temperature. ^f^ Serum vitrified in dextran-based matrix and stored at 37°C.
