# Supplementary material for: Dextranol: An inert xeroprotectant
Source: PLoS One. 2019 Sep 6;14(9):e0222006. doi: 10.1371/journal.pone.0222006 (PMC6730909; doi:10.1371/journal.pone.0222006)
Supplement: S1 Fig — Diminished pellet size shows increased solubility somewhat in the sample stored at room temperature (middle tube) and largely in the sample stored at 37°C (right tube) compared to frozen sample without matrix (left tube). (DOCX) [file pone.0222006.s002.docx]

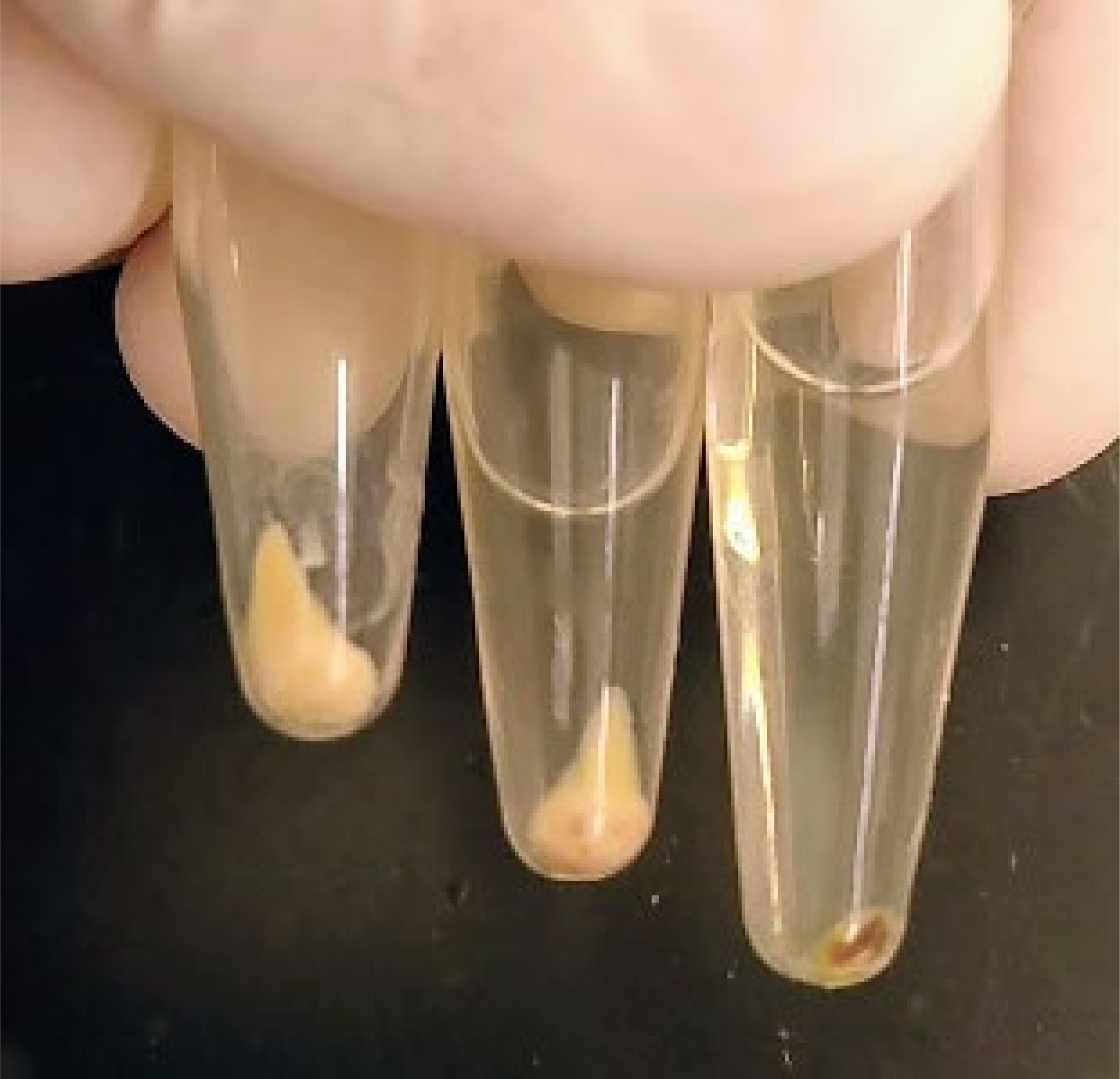


**S1 Figure.** **TCA precipitation of serum proteins stored 35 days in dextran-based matrix.** Diminished pellet size shows increased solubility somewhat in the sample stored at room temperature (middle tube) and largely in the sample stored at 37°C (right tube) compared to frozen sample without matrix (left tube).
