# Supplementary material for: Dextranol: An inert xeroprotectant
Source: PLoS One. 2019 Sep 6;14(9):e0222006. doi: 10.1371/journal.pone.0222006 (PMC6730909; doi:10.1371/journal.pone.0222006)
Supplement: S2 Fig — (A) Proton signals at (6.74 and 6.30 ppm) indicate dextran aldehydes, while (B) loss of these signals shows that aldehydes were completely reduced to alcohols in dextranol. (DOCX) [file pone.0222006.s003.docx]

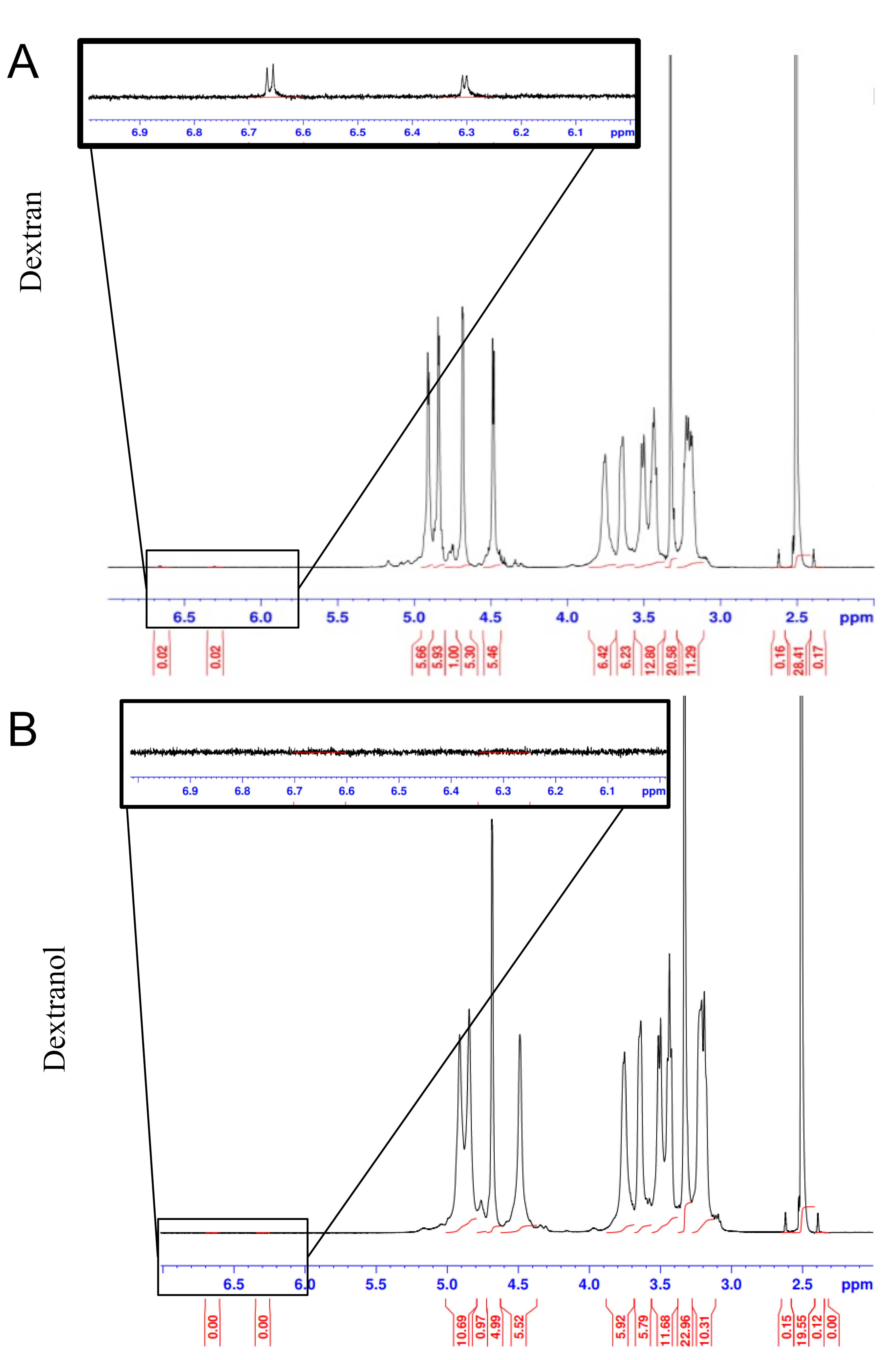


**S2 Figure. H_1_-NMR showing complete reaction and disappearance of protons attached to the anomeric carbon.** (**A**) Proton signals at (6.74 and 6.30 ppm) indicate dextran aldehydes, while (**B**) loss of these signals shows that aldehydes were completely reduced to alcohols in dextranol.
