# Supplementary material for: Dextranol: An inert xeroprotectant
Source: PLoS One. 2019 Sep 6;14(9):e0222006. doi: 10.1371/journal.pone.0222006 (PMC6730909; doi:10.1371/journal.pone.0222006)
Supplement: S3 Fig — DSC traces of serum samples preserved in either dextran (A) or dextranol (B) based matrix after 30 days of storage at room temperature. Solid blue line is DSC data, dashed green line is linear glass-transition fit, dashed red line is the linear fit of liquid region (fit to grey-shaded region), dashed cyan line is the liner fit of the glassy region (fit to green-shaded region). The vertical tan line marks the glass transition temperature (Tg) at 54.7°C (dextran, A) and 53.9°C (dextranol, B). The vertical gold line marks the glass transition onset temperature (Tgon) at 53.2°C (dextran, A) and 53.1°C (dextranol, B). (DOCX) [file pone.0222006.s004.docx]

**
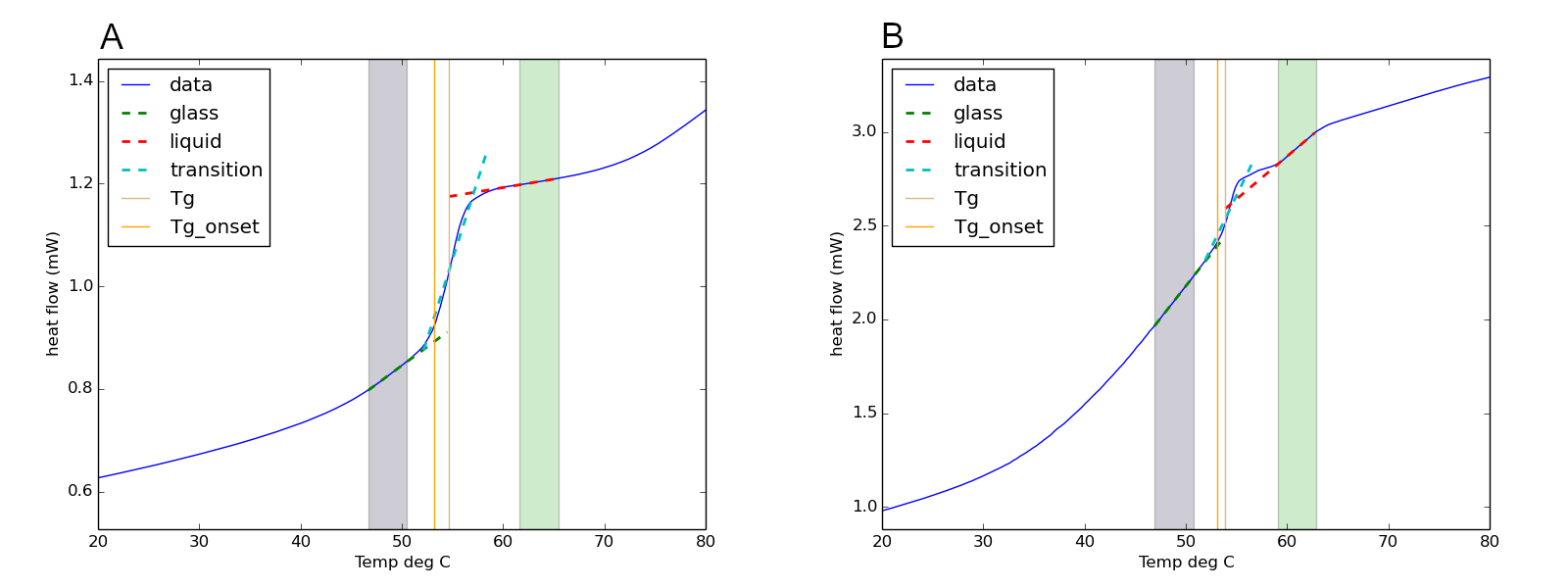
**

**S3 Figure. T_g_ and T_gon_ of serum preserved in either dextran and dextranol.** DSC traces of serum samples preserved in either dextran (**A**) or dextranol (**B**) based matrix after 30 days of storage at room temperature. Solid blue line is DSC data, dashed green line is linear glass-transition fit, dashed red line is the linear fit of liquid region (fit to grey-shaded region), dashed cyan line is the liner fit of the glassy region (fit to green-shaded region). The vertical tan line marks the glass transition temperature (T_g_) at 54.7°C (dextran, **A**) and 53.9°C (dextranol, **B**). The vertical gold line marks the glass transition onset temperature (T_gon_) at 53.2°C (dextran, **A**) and 53.1°C (dextranol, **B**).
