# Supplementary material for: Dextranol: An inert xeroprotectant
Source: PLoS One. 2019 Sep 6;14(9):e0222006. doi: 10.1371/journal.pone.0222006 (PMC6730909; doi:10.1371/journal.pone.0222006)
Supplement: S4 Fig — Serum samples were either fresh, frozen, or vitrified in either a dextran-based or dextranol-based xeroprotectant matrix. Serum was analyzed after 1, 7, 14, 28, 60, and 140 days at 37°C (data from days 1 and 140 in Fig 4). Vitrified samples were reconstituted in PBS. Gel electrophoresis was carried out under both native conditions (A) and denaturing/reducing (B) conditions. A duplicate denatured/reduced gel from the sixty-day old sample was also stained for glycoproteins (C). After two weeks, smearing is visible in samples preserved in dextran, but not in samples preserved in dextranol. (DOCX) [file pone.0222006.s005.docx]

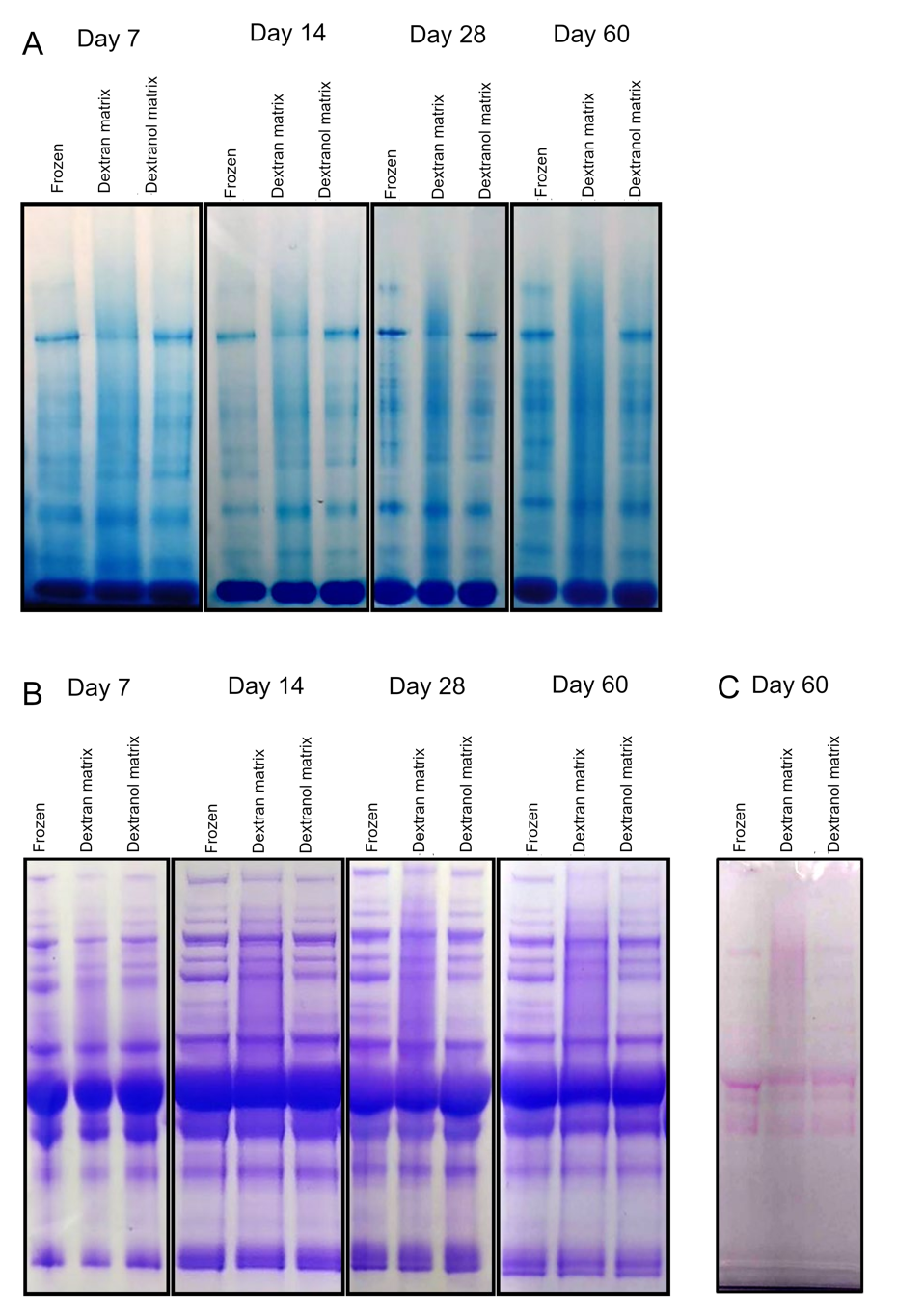


**S4 Figure. Dextranol preserves proteins and prevents smearing compared with dextran.** Serum samples were either fresh, frozen, or vitrified in either a dextran-based or dextranol-based xeroprotectant matrix. Serum was analyzed after 1, 7, 14, 28, 60, and 140 days at 37°C (data from days 1 and 140 in **Fig 4**). Vitrified samples were reconstituted in PBS. Gel electrophoresis was carried out under both native conditions (**A**) and denaturing/reducing (**B**) conditions. A duplicate denatured/reduced gel from the sixty-day old sample was also stained for glycoproteins (**C**). After two weeks, smearing is visible in samples preserved in dextran, but not in samples preserved in dextranol.
