# Supplementary material for: Dextranol: An inert xeroprotectant
Source: PLoS One. 2019 Sep 6;14(9):e0222006. doi: 10.1371/journal.pone.0222006 (PMC6730909; doi:10.1371/journal.pone.0222006)
Supplement: S5 Fig — ELISA analysis of biomarker stability in vitrified human serum stored at high temperature (45°C), preserved in either dextran-based (orange) or dextranol-based matrix (green). Four biomarkers examined were (A) PSA (prostate specific antigen), (B) neuropilin-1, (C) osteopontin, and (D) MMP-7 (matrix-metalloproteinase 7). Serum samples were analyzed immediately after desiccation (day 1), and one week and two weeks after desiccation and storage. Values are normalized to biomarker content in frozen control samples. Error bars are standard deviation of three replicates. (DOCX) [file pone.0222006.s006.docx]

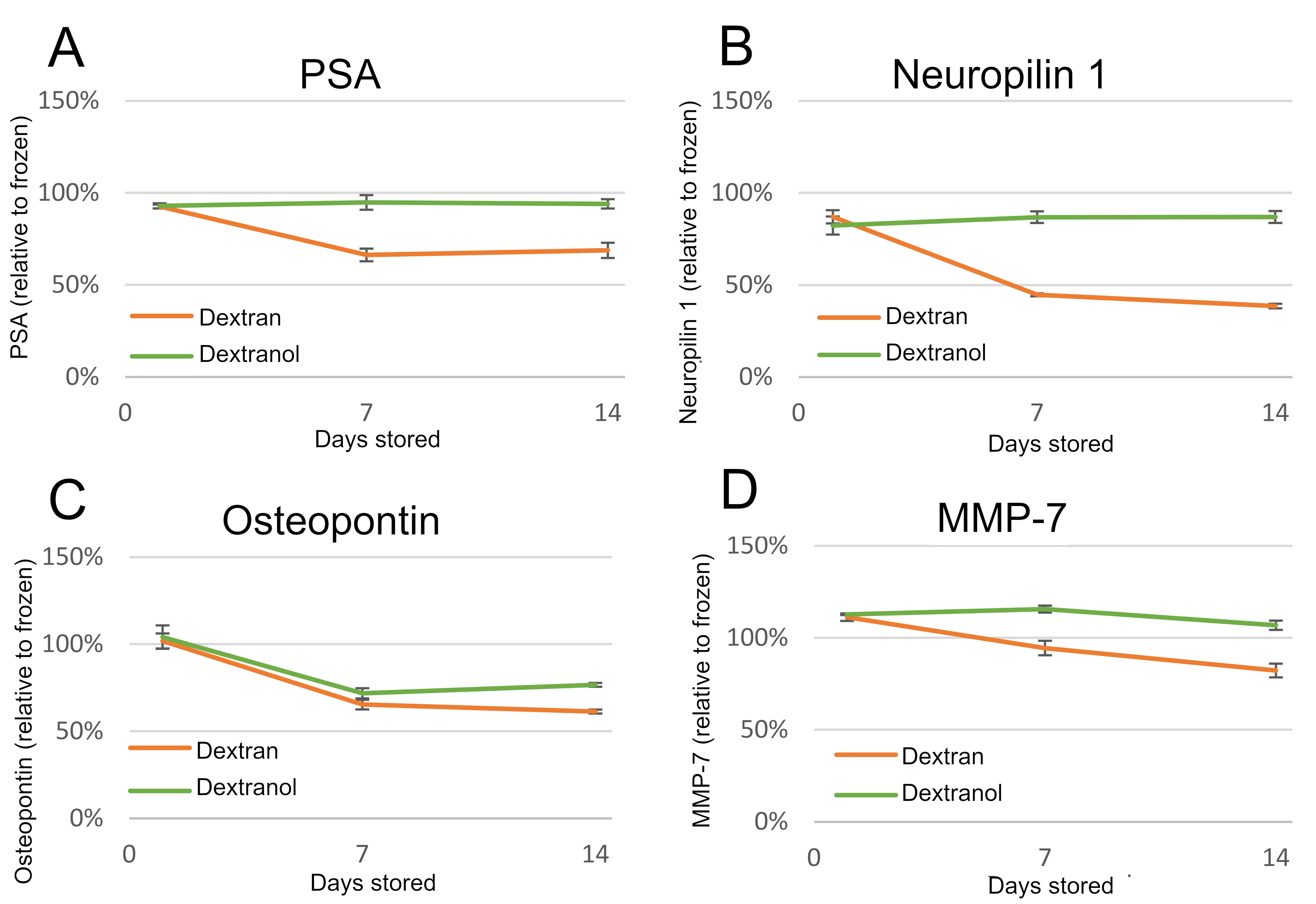


**S5 Figure. Serum biomarker levels are better retained after storage at 45°C when vitrified in dextranol than dextran.** ELISA analysis of biomarker stability in vitrified human serum stored at high temperature (45°C), preserved in either dextran-based (orange) or dextranol-based matrix (green). Four biomarkers examined were (**A**) PSA (prostate specific antigen), (**B**) neuropilin-1, (**C**) osteopontin, and (**D**) MMP-7 (matrix-metalloproteinase 7). Serum samples were analyzed immediately after desiccation (day 1), and one week and two weeks after desiccation and storage. Values are normalized to biomarker content in frozen control samples. Error bars are standard deviation of three replicates.
