# Supplementary material for: Dextranol: An inert xeroprotectant
Source: PLoS One. 2019 Sep 6;14(9):e0222006. doi: 10.1371/journal.pone.0222006 (PMC6730909; doi:10.1371/journal.pone.0222006)
Supplement: S6 Fig — Comparison of lyophilized and frozen samples after indicated storage duration and temperature. Lyophilized samples were preserved with either dextran or dextranol while the frozen sample contained neither xeroprotectants. After reconstitution or thawing, native IgG (green colored area) and soluble large-molecular-weight species (sand colored area) were distinguishable size-exclusion chromatography. (DOCX) [file pone.0222006.s007.docx]

**
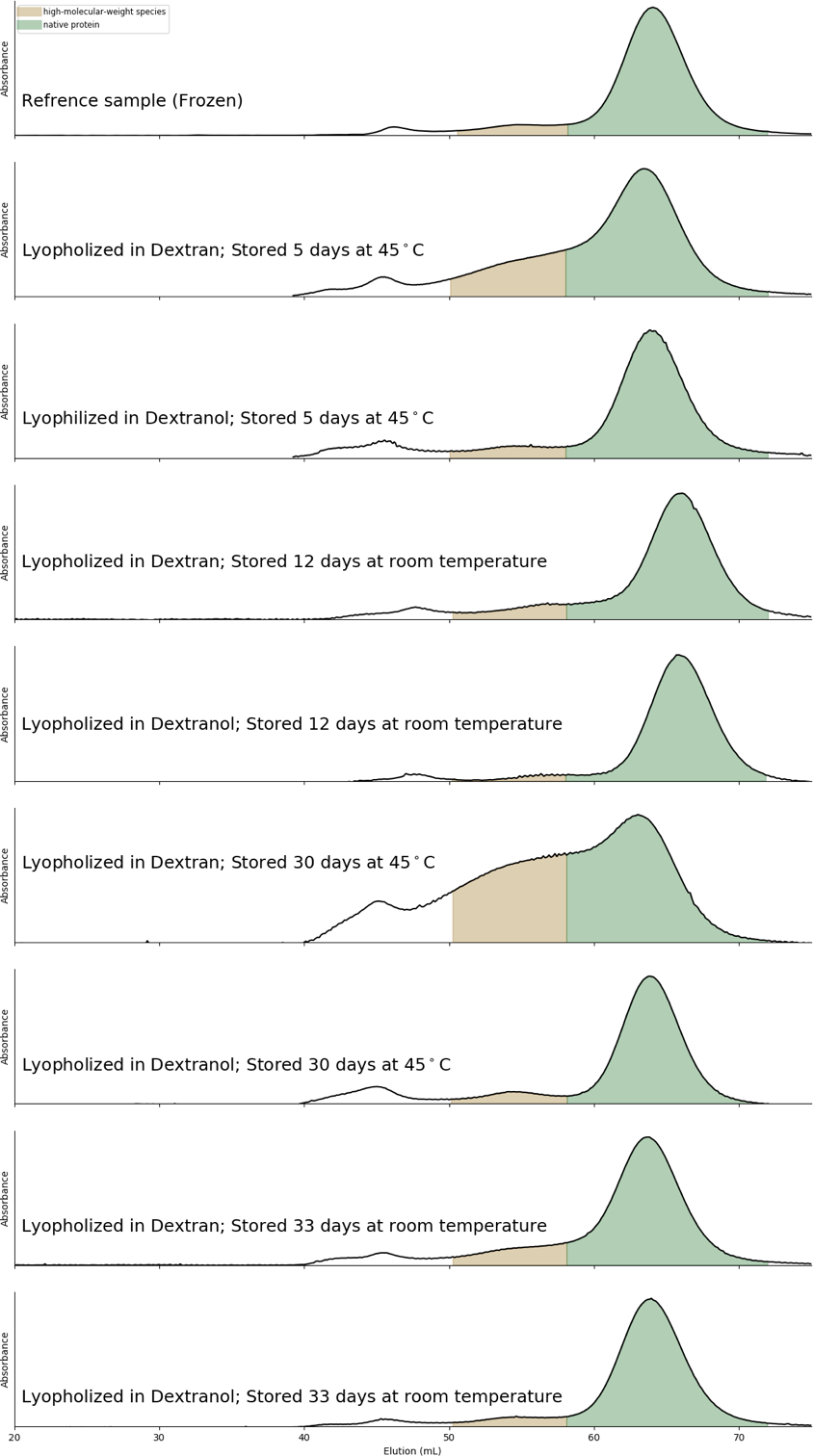
**

**S6 Figure. High-molecular-weight adducts of lyophilized IgG by size-exclusion chromatography at multiple storage time points.** Comparison of lyophilized and frozen samples after indicated storage duration and temperature. Lyophilized samples were preserved with either dextran or dextranol while the frozen sample contained neither xeroprotectants. After reconstitution or thawing, native IgG (green colored area) and soluble large-molecular-weight species (sand colored area) were distinguishable size-exclusion chromatography.
