# Supplementary material for: Dextranol: An inert xeroprotectant
Source: PLoS One. 2019 Sep 6;14(9):e0222006. doi: 10.1371/journal.pone.0222006 (PMC6730909; doi:10.1371/journal.pone.0222006)
Supplement: S7 Fig — MALDI-TOF MS was performed on myoglobin lyophilized with dextran or dextranol (in PBS solution). A clear spectrum for fresh protein in solution (positive control) can be seen in A. Spectra for frozen dextran and dextranol can be seen in B, and C, respectively. The spectra for lyophilized samples stored at 45ºC can be seen in D, and E, respectively. Mass Spectrometry Method: Recombinant human myoglobin (Novus Biologicals) was mixed with dextran/dextranol in PBS solution at final concentration of 6μM and 500μM respectively. 200 μL aliquots were lyophilized and either stored frozen at -20°C or stored at 45°C for three weeks. The frozen and vitrified samples were reconstituted in 100μL DI water post storage. A fresh control samples containing myoglobin in PBS was also prepared. The Center of Mass Spectrometry and Proteomics at the University of Minnesota performed matrix assisted laser desorption/ionization-time of flight (MALDI-TOF) mass spectrometry (MS) on these samples. The samples were prepared using C4 ZipTip protocol prior to loading them on Bruker’s Autoflex speed MALDI-TOF System. The ionization matrix was Sinapinic acid (SA) for the control sample and super-dihydroxybenzonic acid(sDHB) matrix for dextran/dextranol containing samples, where it was found to provide better signal than SA. The data was analyzed using mMass software. (DOCX) [file pone.0222006.s008.docx]

**S7 Figure.** **Mass Spectrometry on Lyophilized Myoglobin with Dextran/Dextranol**. MALDI-TOF MS was performed on myoglobin lyophilized with dextran or dextranol (in PBS solution). A clear spectrum for fresh protein in solution (positive control) can be seen in **A**. Spectra for frozen dextran and dextranol can be seen in **B,** and **C,** respectively. The spectra for lyophilized samples stored at 45ºC can be seen in **D,** and **E,** respectively. Mass Spectrometry Method: Recombinant human myoglobin (Novus Biologicals) was mixed with dextran/dextranol in PBS solution at final concentration of 6µM and 500µM respectively. 200 µL aliquots were lyophilized and either stored frozen at -20°C or stored at 45°C for three weeks. The frozen and vitrified samples were reconstituted in 100µL DI water post storage. A fresh control samples containing myoglobin in PBS was also prepared. The Center of Mass Spectrometry and Proteomics at the University of Minnesota performed matrix assisted laser desorption/ionization-time of flight (MALDI-TOF) mass spectrometry (MS) on these samples. The samples were prepared using C4 ZipTip protocol prior to loading them on Bruker’s Autoflex speed MALDI-TOF System. The ionization matrix was Sinapinic acid (SA) for the control sample and super-dihydroxybenzonic acid(sDHB) matrix for dextran/dextranol containing samples, where it was found to provide better signal than SA. The data was analyzed using mMass software.
